# Anti‐Osteoporosis Medications Associated with Decreased Mortality after Hip Fracture

**DOI:** 10.1111/os.12517

**Published:** 2019-08-20

**Authors:** Pei‐wen Wang, Yi‐zhong Li, Hua‐feng Zhuang, Hai‐ming Yu, Si‐qing Cai, Hao Xu, Zhen‐hui Chen, Jin‐kuang Lin, Xue‐dong Yao

**Affiliations:** ^1^ Department of Orthopaedics The Second Affiliated Hospital of Fujian Medical University Quanzhou China; ^2^ Department of Radiology The Second Affiliated Hospital of Fujian Medical University Quanzhou China

**Keywords:** Anti‐osteoporosis medication, Bisphosphonate, Hip fracture, Mortality

## Abstract

**Objective:**

To study the effect of anti‐osteoporosis therapies on mortality after hip fracture.

**Methods:**

This retrospective study was carried out in the Second Affiliated Hospital of Fujian Medical University and enrolled 690 patients 50 years of age and older who were admitted with hip fractures between 2010 and 2015. The patients were followed in 2017: 690 patients aged was from 50 to 103 years. There were 456 women and 234 men. There were 335 patients with fractures of the femoral neck and 355 patients with intertrochanteric fractures of the femur. There were 444 (64.35%) patients who also had internal diseases. The Charlson comorbidity index was 0–6. The anti‐osteoporosis medications were classified into no anti‐osteoporosis medication, calcium + vitamin D supplementations, non‐bisphosphonate medication, and bisphosphonate medication. The physicians followed the patients or family members by personal visit and telephone. Multivariable Cox regression analyses were done with known risk factors for mortality of hip fracture, such as gender, age, number of combined internal diseases, fracture type, place of residence, and Charlson comorbidity index, to show which anti‐osteoporosis medications had significant effects on mortality after adjustment for these variables.

**Results:**

Out of 690 patients with hip fractures, 149 patients received no anti‐osteoporosis medication, 63 patients received calcium +vitamin D supplementations, 398 patients received non‐bisphosphonate medication, and 80 patients received bisphosphonate medication. The patients were followed between 7 months and 52 months, with the average of 28.53 ± 9.75 months. A total of 166 patients died during the follow‐up period. Of 166 deaths, 43 occurred within 3 months, 65 within 6 months, and 99 within 1 year after the hip fracture. In this study, fracture type, place of residence, and Charlson comorbidity index were not associated with the mortality, and the male gender, age > 75 years, and ≥ 2 combined internal diseases were the independent factors for deaths post‐hip fracture. The cumulative mortality was 36.24% in the patients receiving no anti‐osteoporosis medication. The hazard ratio for mortality after hip fracture with bisphosphonate medication, non‐bisphosphonate medication, and calcium/vitamin D supplementation was 0.355 (95% *CI*, 0.194–0.648), 0.492 (95% *CI*, 0.347–0.699) and 0.616 (95% *CI*, 0.341–1.114), respectively, as compared with no anti‐osteoporosis group. Bisphosphonate and non‐bisphosphonate medications for osteoporosis were significantly associated with the reduction of cumulative mortality post‐hip fracture (*P* < 0.01).

**Conclusions:**

Bisphosphonate and non‐bisphosphonate medications for osteoporosis were significantly associated with decreased mortality after fragility hip fracture.

## Introduction

Hip fragility fractures including femoral neck fracture and femoral intertrochanteric fractures are common in the older population. It is a common and severe complication of osteoporosis. Hip fragility fractures are more common in females than males. The number of hip fractures is expected to increase from 1.5 million in 2000 to 6.3 million in 2050 worldwide^1^. Hip fractures in China ascended at a rate of approximately 10% each year in 2002–2006^2^. The incidence rate of hip fractures increases with increasing age for both women and men aged 50 years or older. Fragility fractures are significantly associated with osteoporosis. Osteoporosis is characterized by low bone mass. The bone mineral density (BMD) measured by dual‐energy X‐ray absorptiometry (DXA) is the “gold standard” for diagnosis of osteoporosis and shows low bone mineral density and poor hip structure in patients with fragility hip fractures^3^. Low BMD and bone quality increase the risk of fracture and prevalent fractures significantly increase the risk of future fractures. It is necessary to break the fragility fracture cycle. Pharmacotherapy is a key measure for increase in BMD and prevention of future fractures. Anti‐osteoporosis therapy for patients with fragility hip fractures has been recommended by European guidance for the diagnosis and management of osteoporosis in postmenopausal women and UK clinical guidelines for the prevention and treatment of osteoporosis^4^, ^5^. A daily calcium intake of between 700 and 1200 mg and a daily dose of 800 IU of cholecalciferol are advised. Bisphosphonates, calcitonin, calcitriol, and alfacalcidol are recommended for osteoporosis treatment and bisphosphonates are the first‐line drugs in China.

Orthopaedic surgeons are increasingly paying attention to osteoporosis and providing anti‐osteoporosis therapy to patients after the surgical management of hip fractures during the period of hospitalization. Fragility hip fractures are associated with high morbidity and mortality^6^, ^7^. The increased mortality is significant within the first year after the fracture and extends beyond 10 years^8^. The high mortality in patients with hip fractured is associated with many factors such as age, gender, co‐morbidity disease, fracture type, surgery type, the skills of the surgeons, and the medical care systems^9^, ^10^. The risk of post‐fracture mortality is also significantly higher for persons who have a subsequent fracture and do not take anti‐osteoporosis pharmacologic therapy following the fracture^8^, ^11^. How can the risk of mortality be reduced in patients with hip fractures? There is no consensus regarding the best treatment factors to optimize outcomes after hip fracture surgery. Early surgery and early mobilization are considered the key factors to reduce subsequent mortality risk^12^, ^13^. Patients with hip fractures should take measures to prevent secondary fractures. Anti‐osteoporosis medications are important and effective for prevention of future fractures.

Anti‐osteoporosis therapy has been proven to reduce the risk of subsequent fractures and to improve the functional outcomes in patients with hip fractures, leading to the hypothesis that drug therapy for osteoporosis can reduce the risk of death in patients with hip fragility fractures. Bisphosphonates are recommended and widely used for osteoporotic patients. Bisphosphonates can effectively increase the BMD of the lumbar spine, the femoral neck, and the total hip. Bisphosphonates not only significantly reduce the risk of vertebral fractures but also significantly reduce the risk of hip fractures. Zoledronic acid is an intravenous, highly potent bisphosphonate. Zoledronate (5 mg, administered once yearly) is safe and suitable for in‐hospital initiation of anti‐osteoporosis therapy in patients with hip fractures after surgery for fracture management, although there are some potential acute phase symptoms after intravenous infusion. Zoledronate does not appear to delay human fracture healing when the use of zoledronate is initiated following the acute fracture. Zoledronate has proven to significantly increase the BMD of hip and lumbar spine, to reduce the risk of new clinical fractures by 35%, and to reduce the risk of death in patients with hip fractures by 28% during a 3 year period in the Horizon Recurrent Fracture Trial^14^.

Whether zoledronate is associated with decreased mortality in ordinary clinical practice is still unclear and whether other anti‐osteoporosis medications including non‐bisphosphonate medication and supplementation of calcium and vitamin D have similar results is also unclear. In this retrospective study, we enrolled 690 patients with hip fractures to: (i) determine the mortality in patients with hip fractures who were admitted to the Second Affiliated Hospital of Fujian Medical University; (ii) understand the medications for osteoporosis; and (iii) study the impact of four kinds of anti‐osteoporosis medications, including no anti‐osteoporosis medication, calcium + vitamin D supplementations, non‐bisphosphonate medication, and bisphosphonate medication on mortality post‐hip fracture.

## Patients and Methods

### *Study Population*

This retrospective study observes the effect of anti‐osteoporosis medications on cumulative mortality after fragility hip fractures. A total of 1211 patients with hip fragility fractures who were 50 years of age and older were consecutively admitted to the Second Affiliated Hospital of Fujian Medical University in China from January 2010 to December 2015.

#### *Inclusion and Exclusion Criteria*

The inclusion criteria in our study were: (i) patients admitted with new hip fragility fractures and patients' aged ≥50 years; (ii) patients who received no anti‐osteoporosis medication, or calcium + vitamin D supplementations, or non‐bisphosphonate medication, or bisphosphonate medication for osteoporosis; (iii) mortality in the patients with different medications for osteoporosis used for comparison; (iv) the impact of four kinds of anti‐osteoporosis medications on mortality.

Exclusion criteria were as follows: (i) pathological fractures caused by malignant tumors; (ii) patients with total hip or femoral head replacement; and (iii) high‐energy fractures and age below 50 years.

#### *Follow‐up Method*

The patients' medical records were retrospectively reviewed. The patients admitted for brittle hip fractures from January 2010 to December 2012 were followed up in 2013 and followed up again in 2017, and the patients admitted from January 2013 to December 2015 were followed up in 2017.

The physicians followed up the patients or the family members who lived with the patients before death by personal visit and telephone in this study. Time to death was calculated from admission to possible event. Survival time was calculated in months. The date of death or the last interview with the patients or the family members was used to determine the end of follow up. A total of 521 patients were lost to follow up because of failure to make contact with patients or their relatives by telephone and letters.

#### *General Information*

A total of 690 patients aged ≥50 years with hip fragility fractures were enrolled in this retrospective research. The patients were aged from 50 to 103 years, with the average of 78.08 ± 9.7 years. There were 456 women and 234 men. There were 323 patients living in cities and 367 patients living in the countryside. There were 335 patients with fractures of the femoral neck and 355 with intertrochanteric fractures of the femur. There were 444 (64.35%) patients with internal diseases and 231 (33.48%) with two or more types of internal disease (34%). The main combined diseases were cardiovascular and cerebrovascular diseases, diabetes mellitus, respiratory disease, liver cirrhosis, and renal insufficiency. The Charlson comorbidity index (CCI) was 0–6.

The medications for osteoporosis were indicated in all patients. The bisphosphonates were not recommended to the patients with creatinine clearance <35 mL/min. The medications administered to the patients depended on the consensus between surgeons and patients, which was influenced by surgeons' awareness of osteoporosis knowledge, patients' perception of bone health, understanding of drug safety, medical insurance, and financial ability.

### *Classification of Medications for Osteoporosis*

According to the medications for osteoporosis during hospitalization and after discharge, the anti‐osteoporosis medications were classified into no anti‐osteoporosis medication, calcium + vitamin D supplementations, non‐bisphosphonate medication, and bisphosphonate medication.

#### *Anti‐Osteoporosis Medications*

(i) No anti‐osteoporosis medication was defined as patients who have no calcium + vitamin D supplementations and any drug for osteoporosis; (ii) calcium + vitamin D supplementations were defined as patients who only had calcium + vitamin D supplementations; (iii) non‐bisphosphonate medication was defined as patients who were administered with calcitonin and/or calcitriol or alfacalcidol (the patients in this group also had calcium and /or vitamin D supplementations); and (iv) bisphosphonate medication was defined as patients who had one of the bisphosphonates for osteoporosis (the patients in this group also had calcium and/or vitamin D supplementations).

#### *Medications Methods*

The patients took the medications for osteoporosis within 2 weeks after the admission. The daily supplementation of calcium was 500–1000 mg and the daily dose of vitamin D was 800–1200 IU. The daily dose was 0.25–0.5 μg of calcitriol or 0.5–1 μg of alfacalcidol, 50 U of calcitonin intramuscular injection or 200 U of calcitonin nasal spray; 5 mg of zoledronic acid was the only used bisphosphonate. The patients were advised to see doctors for osteoporosis every 3 months after discharge.

All‐cause mortality and the relative risk in the patients with four kinds of medication were identified. The impact of anti‐osteoporosis medications on mortality was analyzed.

### *Statistical Analysis*

A summary of the data was presented as mean, standard deviation, and percentage.

For comparisons of patients' age and CCI in the no anti‐osteoporosis group with the other three groups, *t*‐tests were used. For comparisons of gender, patients with ≥2 combined internal disease, and mortality post‐hip fracture in the no anti‐osteoporosis group with other three group, χ^2^‐tests were used.

To determine which of the anti‐osteoporosis medications had a significant effect on mortality, known risk factors for mortality of hip fracture were included in the multivariate Cox proportional hazard regression analysis. All statistical analyses were performed using SPSS version 25.0 software. *P* < 0.05 was considered as statistically significant.

## Results

### *Anti‐Osteoporosis Medications*

(i) A total of 149 patients received no anti‐osteoporosis medication; (ii) 63 patients received calcium +vitamin D supplementations and most patients reported that medication compliance was 80% or higher; (iii) 398 patients received non‐bisphosphonate medication and most patients reported that medication compliance was 60% or higher; and (iv) 80 patients received bisphosphonate medication and took annual infusions of 5 mg of zoledronic acid 1–3 times. There were no severe adverse events such as osteonecrosis of the mandible and atypical femoral fractures caused by bisphosphonates.

### *Baseline Characteristics of Patients in Four Groups*

Patients' age in non‐bisphosphonate medication and bisphosphonate medication groups was significantly greater than no anti‐osteoporosis medication group. CCI in non‐bisphosphonate medication and bisphosphonate medication groups was significantly lower than in the no anti‐osteoporosis medication group.

There were more male patients and patients with ≥2 combined internal diseases in the no anti‐osteoporosis group than in other three groups. Mortality post‐hip fracture in the no anti‐osteoporosis group was significantly higher than in the other three groups.

### *All‐Cause Mortality*

A total of 690 patients with hip fractures were followed between 7 months and 52 months, with the average of 28.53 ± 9.75 months; 166 patients died during the follow‐up period and the cumulative mortality after hip fracture was 24.06%. There were 69 men and 97 women among these 166 deaths and the cumulative mortality was 29.49% in the male patients and 21.27% in the female patients. Of 166 deaths, 43 occurred within 3 months, 65 within 6 months, and 99 within 1 year after the hip fracture. The common causes of mortality were cardiovascular or cerebrovascular events, pneumonia, and cancer.

### *Impact of Anti‐Osteoporosis Medications on the Mortality*

#### *Mortality of Four Anti‐Osteoporosis Medications*

There were 54 (36.24%) deaths in the patients receiving no anti‐osteoporosis medication, 14 (22.22%) deaths in the patients receiving calcium +vitamin D supplementations, 84 (21.11%) deaths in the patients receiving non‐bisphosphonate medication, and 14 (17.5%) deaths in the patients receiving bisphosphonate medication. The differences of the four groups in mortality were of statistical significance (*P* < 0.05, Table 1). No anti‐osteoporosis medication resulted in an obvious increase (almost 150% to 2 times) in the mortality compared with calcium +vitamin D supplementations, non‐bisphosphonate medication, and bisphosphonate medication.

**Table 1:** Baseline characteristics of hip fracture patients in four medication groups

Characteristic	No anti‐osteoporosis group	Calcium + vitamin D supplementations	Non‐bisphosphonate medication	Bisphosphonate medication
Patients number	149	63	398	80
Age (years, mean ± SD)	76.20 ± 9.92	75.65 ± 9.83	78.67 ± 10.25	79.76 ± 10.32
Male (cases [%])	69 (46.31)	20 (31.75)	129 (32.41)	16 (20.00)
Female (cases [%])	80 (53.69)	43 (68.25)	269 (67.59)	64 (80.00)
Living place (city/countryside)	68/81	22/41	182/216	51/29
Combined internal disease (cases [%])	112 (75.17)	38 (60.32)	248 (62.31)	46 (57.50)
<2 combined internal disease (cases [%])	39 (26.17)	21 (33.33)	123 (30.90)	30 (37.50)
≥2 Combined internal disease (cases [%])	73 (48.99)	17 (26.98)	125 (31.41)	16 (20.00)
Renal impairment (cases [%])	4 (2.68)	3 (4.76)	0 (0)	0 (0)
CCI	0.725	0.429	0.462	0.313
Fracture of femoral neck (cases [%])	59 (39.60)	30 (47.62)	199 (50.00)	47 (58.75)
Intertrochanteric fracture (cases [%])	90 (60.40)	33 (52.38)	199 (50.00)	33 (41.25)
Mortality post‐hip fragility fracture (cases [%])	54 (36.24)	14 (22.22)	84 (21.11)	14 (17.50)

#### *Multivariate Cox Regression Analysis*

**Figure 1:**
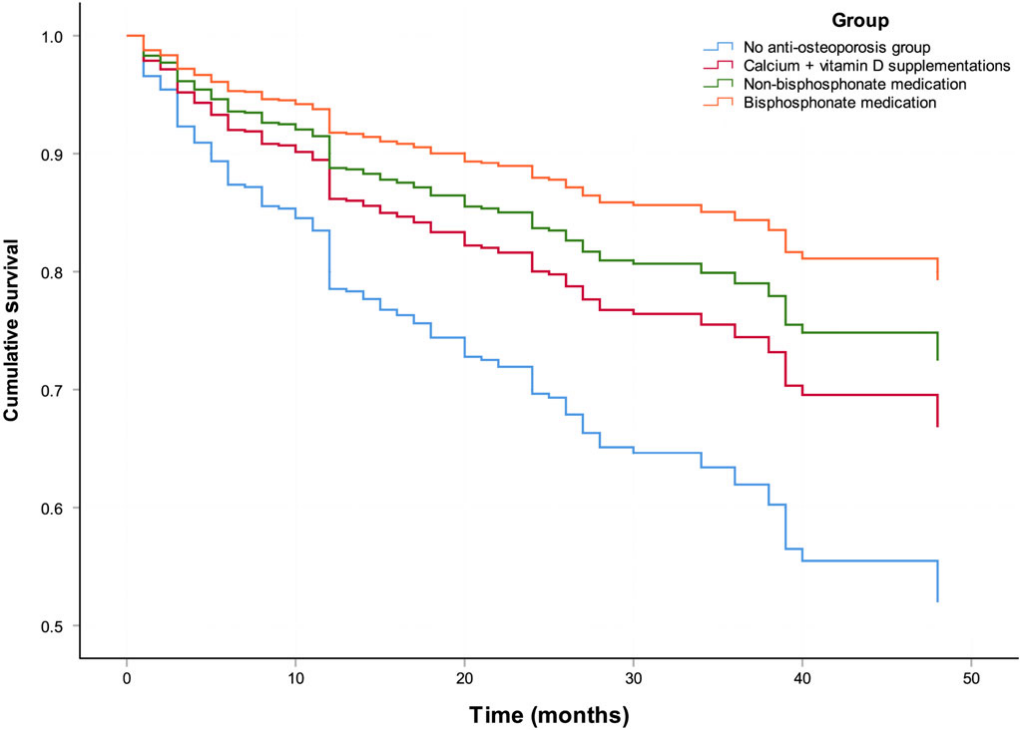
Adjusted survival curve for the patients with hip fracture according to different anti‐osteoporosis medications (multivariable Cox regression analyses adjusted with gender, age, and combined disease number).

The multivariate Cox proportional hazards model was applied to estimate the effects of factors on survival time. The factors evaluated in this model included medications, age, gender, number of combined internal diseases, fracture type, place of residence, and CCI. In this study, fracture type, place of residence, and CCI were not associated with mortality, and the male gender, age > 75 years, and ≥ 2 combined internal diseases were the independent factors for deaths post‐hip fracture (Table 2). The hazard ratio for mortality after hip fracture with bisphosphonate medication, non‐bisphosphonate medication and calcium/vitamin D supplementation was 0.355 (95% *CI*, 0.194–0.648), 0.492 (95% *CI*, 0.347–0.699), and 0.616 (95% *CI*, 0.341–1.114), respectively, as compared with the no anti‐osteoporosis group (Table 2). After the adjustment of these risk factors, multivariable Cox regression analyses confirmed that non‐bisphosphonate and bisphosphonate medication for osteoporosis were significantly associated with the reduction of cumulative mortality post‐hip fracture (*P* < 0.01). There was no significant difference in cumulative mortality between non‐bisphosphonate and bisphosphonate medication (*P* > 0.05). No significant association with survival was found between the calcium + vitamin D supplementations group and the no anti‐osteoporosis group (*P* = 0.097; *HR* = 0.606, 95%*CI*, 0.335–1.095) (Fig. 1).

**Table 2:** Multivariate Cox regression analysis of mortality in hip fracture patients

Variables	*HR*	95% *CI*	*P‐*value
Gender			
Female	1.000 (reference)		
Male	1.679	1.225–2.300	0.001
Age			
<75 years	1.000 (reference)		
≥75 years	4.822	3.014–7.717	<0.001
Number of combined internal disease			
<2	1.000 (reference)		
≥ 2	1.554	1.132–2.133	0.006
Groups			
No anti‐osteoporosis group	1.000 (reference)		
Calcium + vitamin D supplementations	0.616	0.341–1.114	0.109
Non‐bisphosphonate medication	0.492	0.347–0.699	<0.001
Bisphosphonate medication	0.355	0.194–0.648	0.001

*CI*, confidence interval; *HR*, hazard ratio

## Discussion

In this study, the cumulative mortality after hip fragility fracture was 24.06% during the follow‐up period. The fracture type, place of residence, and CCI were not associated with the mortality, and the male gender, age > 75 years, and ≥ 2 combined internal diseases were the risk factors for deaths post‐hip fracture in this study. The cumulative mortality in the patients receiving no anti‐osteoporosis medication was as high as 36.24% during follow‐up. Our study confirmed that non‐bisphosphonate and bisphosphonate medications for osteoporosis still significantly reduced the cumulative mortality after the adjustment of the factors of gender, age, and number of combined internal diseases and that zoledronic acid had the most effective impact on the reduction of mortality after hip fracture.

### *High Mortality of Hip Fracture*

Hip fragility fractures are a leading cause of mortality in patients aged ≥65 years^15^. The first year mortality post‐fracture was five times higher in men and three times higher in women^16^, and reached 16% to3 0%, respectively^6^, ^7^. There are many risk factors related with the death of patients with hip fractures. Sheehan *et al*.^17^ synthesized the evidence from 56 articles and identified 35 patient factors and 9 medical system factors of mortality post‐hip fracture. The main patient factors include age, sex, comorbidity, heart disease, pneumonia, and renal failure. The main medical system factors include hospitalization delay, surgical delay, anesthetic type, and intensive care admission. Therefore, we cannot be sure whether intervening on some of these factors may produce the expected results. In fact, it is difficult to reduce the all‐cause mortality after hip fracture.

### *Impact of Anti‐Osteoporosis Medications on Mortality of Hip Fracture*

Fragility fractures lead to excess mortality and subsequent fractures, including hip fractures and vertebral fractures. Several anti‐osteoporosis medications have proved to effectively reduce the fracture risk^18^. Prevention of new fractures is important for patients with fragility hip fractures. Low BMD and prior fracture significantly increase the risk of future fractures, including contralateral hip fractures and vertebral fractures. The osteoporosis pharmacotherapy is one of the most important methods in preventing new fractures after management of hip fractures. Bisphosphonates including alendronate, risedronate, and zoledornic acid are recommended as first‐line drugs in the treatment of osteoporosis, and non‐bisphosphonate drugs, including calcitonin, calcitriol, and alfacalcidol are also recommended in many guidelines. Zoledronic acid (5 mg) was given to patients with hip fractures during the period of hospitalization and ensured the compliance of patients. Zoledronic acid significantly reduced new clinical vertebral fractures and clinical non‐vertebral fractures^14^, and improved functional outcomes with good results for the Harris hip score and the Merle d'Aubigné scale from the 6th week to the 12th month after surgical management of hip fracture^19^. It is surprising that anti‐osteoporosis medications may reduce the all‐cause mortality of patients with hip fractures. Lyles *et al*.^14^ first reported in 2007 that zoledronic acid reduced the mortality in the patients with hip fractures from 13.3% to 9.6% compared with the placebo group in the 3 year Recurrent Fracture Trial. Cengiz *et al*.^19^ reported in 2016 that 5 mg of zoledronic acid significantly reduced the first year mortality in the patients with intertrochanteric femoral fractures from 34.5% in the control group to 14.3% in the treatment group. Beaupre *et al*.^20^ reported in 2011 that oral bisphosphonates (alendronate 70 mg or risedronate 35 mg weekly) reduced the mortality after hip fracture from 16% in non‐ bisphosphonate users to 7% in bisphosphonate users. Su *et al*.^21^ reported in 2015 that postmenopausal women treated with raloxifene had a lower mortality rate than those who did not receive treatment after vertebroplasty. In our study, we confirmed that bisphosphonate medication and non‐bisphosphonate medication were significantly associated with decreased mortality post‐hip fracture, and that after the adjustment of the risk factors of gender, age, and combined internal disease number, non‐bisphosphonate and bisphosphonate medications were still significantly associated with the reduction of cumulative mortality post‐hip fracture. Non‐bisphosphonate medication was given to many patients in this study because calcitonin, calcitriol, and alfacalcidol were recommended by Chinese guidelines. Oral bisphosphonates were not recommended to our patients with hip fractures because oral bisphosphonates have stringent and complex requirements for administration and poor adherence, which significantly reduces the anti‐fracture efficacy. Zoledronate infusion once a year is suitable for initiation of osteoporosis medication in patients with hip fracture and ensures that patients will have a full treatment effect for 12 months. Zoledronate infusion is generally well tolerated, although the adverse events of headache, pyrexia, musculoskeletal pain, fever, and flu‐like illness were common within 3 days after an infusion.

### *Possible Reasons for Decreasing Mortality*

The anti‐osteoporosis medications were the independent factors for reducing mortality post‐hip fracture. The possible reasons for decreasing mortality after hip fracture are as follows. First, zoledronic acid reduces the risk of subsequent fractures, including hip and vertebral fractures, which lead to excess mortality, and improves functional outcomes of the patients with hip fractures. Second, bisphosphonates improve cardiovascular ischemia, and reduce the risk of acute myocardial infarction and deadly cardiac arrhythmias^22^, ^23^. Third, bisphosphonates and vitamin D may take part in the immune responses to serious infections. Studies have confirmed that bisphosphonates target B cells to enhance humoral immune responses and that vitamin D supplementation prevents acute respiratory tract infections^24^, ^25^. Fourth, low 25‐hydroxy‐vitamin D concentration is associated with an increased risk of cardiovascular disease (CVD), including acute myocardial infarction, coronary heart disease, stroke, and CVD mortality^26^, ^27^. Vitamin D and analogues can elevate the serum level of 25‐hydroxy‐vitamin D. Fifth, Vitamin D and analogues can significantly reduce the risk of fall and new fractures. Finally, calcitonin and calcitriol significantly reduce the risk of vertebral fractures, which increase the risk of death^28^.

### *Limitations*

This retrospective study has several limitations. First, we only successfully followed the 57% of the patients with hip fractures and it was a retrospective and unrandomized study. Second, we did not include all factors in our study that might influence the prognosis of the patients with hip fractures, such as the process of orthopaedic surgery, the skills of the surgeons, and the medical care systems. However, this is an encouraging result for anti‐osteoporosis medication after hip fracture for the “real world” clinical practice perspective.

### *Conclusion*

Anti‐osteoporosis medications are needed for the patients with hip fractures. Bisphosphonate and non‐bisphosphonate medications for osteoporosis were significantly associated with the reduction of cumulative mortality after hip fragility fracture.
